# Impact of time to initiation of radiotherapy on survival after resection of newly diagnosed glioblastoma

**DOI:** 10.1186/s13014-019-1272-6

**Published:** 2019-04-29

**Authors:** Sotirios Katsigiannis, Boris Krischek, Stefanie Barleanu, Stefan Grau, Norbert Galldiks, Marco Timmer, Christoph Kabbasch, Roland Goldbrunner, Pantelis Stavrinou

**Affiliations:** 1 Department of Neurosurgery, University Hospital of Bochum, In der Schornau Str. 23-25, 44892 Bochum, Germany; 2 Department of Neurosurgery, University Hospital Cologne, Kerpener Str. 62, 50937 Cologne, Germany; 3 Department of Neurology, University Hospital Cologne, Kerpener Str. 62, 50937 Cologne, Germany; 4 Institute of Neuroscience and Medicine, Research Center Juelich, Juelich, Germany; 5 Center of Integrated Oncology (CIO), Universities of Cologne and Bonn, Cologne, Germany; 6 Department of Neuroradiology, University Hospital Cologne, Kerpener Str. 62, 50937 Cologne, Germany

**Keywords:** Glioblastoma, Timing of radiotherapy, Prognostic factors, Survival, Progression free survival

## Abstract

**Background and purpose:**

To evaluate the effect of timing of radiotherapy (RT) on survival in patients with newly diagnosed primary glioblastoma (GBM) treated with the same therapeutical protocol.

**Materials and methods:**

Patients with newly diagnosed primary GBM treated with the same therapeutical scheme between 2010 and 2015 in our institution were retrospectively reviewed. The population was trichotomized based on the time interval from surgery till initiation of RT (< 28 days, 28–33 days, > 33 days). Kaplan-Meier and Cox regression analyses were used to compare progression free survival (PFS) and overall survival (OS) between the groups. The influence of various extensively studied prognostic factors on survival was assessed by multivariate analysis.

**Results:**

One-hundred-fifty-one patients met the inclusion criteria. Between the three groups no significant difference in PFS (*p* = 0.516) or OS (*p* = 0.902) could be demonstrated. Residual tumor volume (RTV) and midline structures involvement were identified as independent prognostic factors of PFS while age, O-6-Methylguanine Methyltransferase (MGMT) status, Ki67 index, RTV and midline structures involvement represented independent predictors of OS. Patients starting RT after a prolonged delay (> 48 days) exhibited a significantly shorter OS (*p* = 0.034).

**Conclusion:**

Initiation of RT within a timeframe of 48 days is not associated with worsened survival. A prolonged delay (> 48 days) may be associated with worse OS. RT should neither be delayed, nor forced, but should rather start timely, as soon as the patient has recovered from surgery.

## Introduction

Glioblastoma (GBM) is both the most common and lethal primary brain tumor in adults [1, 2]. The standard of care for patients with newly diagnosed GBM comprises maximum safe resection of the tumor followed by radiotherapy (RT) with concomitant and adjuvant temozolomide (TMZ) chemotherapy [3, 4]. Despite this multimodal therapeutical approach, the median overall survival time is approximately 15–17 months [5, 6]. Extensively studied prognostic factors of survival include age, Karnofsky Performance Score (KPS), extent of tumor resection (EOR), residual tumor volume (RTV), O-6-Methylguanine Methyltransferase (MGMT) promoter methylation status, and Ki-67 expression [7–12].

The impact of timing of RT initiation on survival after surgical resection remains controversial [13–18]. From a biological point of view, there are arguments to support an early as well as a late initiation of RT: οn the one hand, an early start of RT could have a negative impact on survival due to reduced radiosensitivity secondary to postoperative hypoxia or due to the “second-impact” effect leading to a further deterioration of the clinical condition of an already compromised patient [19, 20]. On the other hand, a similar case can be made for a delayed initiation of RT: some GBM exhibit increased growth rates and a delayed RT fails to take advantage of this increased radiosensitivity [21]. Studies addressing the effect of timing of RT on survival suffer from considerable bias, such as inhomogeneous patient populations, inhomogenous treatment protocols or disregard for tumor location, EOR, and RTV. In those few studies where the EOR was taken into consideration, no accurate volumetric analysis was performed. Moreover, only two studies took the MGMT status or the Ki-67 proliferation index into consideration [15, 22].

The purpose of this study was to evaluate the timing of RT as a prognostic factor on a homogeneous patient population with only newly diagnosed primary GBM treated with the same treatment scheme, also taking important prognostic factors such as tumor location, MGMT methylation status, EOR and RTV into account.

## Materials and methods

### Patient population

We retrospectively analyzed all adult patients with a newly diagnosed primary GBM treated according to the EORTC 22981/26981 protocol [3] at the Brain Tumor Center of the University Hospital of Cologne, Germany between 2010 and 2015. Neuropathological diagnosis was performed in accordance with the 2016 WHO classification and patients with a mutation encoding for the isocitrate dehydrogenase (IDH) were not included, since they most probably represented secondary GBM with a completely different prognosis [23, 24].

Medical records were reviewed for clinical variables such as age, gender, comorbidities, presenting symptoms, intraoperative parameters, hospital stay, postoperative course, and KPS.

Deep-seated tumors or tumors in highly eloquent areas not amenable to complete resection were excluded from the study. Eloquence of the tumor location was assessed using preoperative transcranial magnetic stimulation, allowing exact mapping of the somatosensory and motor areas and identification of the exact relationship between the glioblastoma and the critical brain regions [25, 26]. Patients with infratentorial or multicentric tumors, as well as patients who received biopsy only or had tumor-debulking surgery without the goal of gross total resection were also excluded (Table 1).

**Table 1:** Study exclusion criteria

Exclusion criteria	
Age ≤ 18 years	
IDH mutated glioblastomas	
Tumors not amenable to complete resection/ tumor debulking surgery	
Biopsy only	
Multicentric lesions	
Timing of radiation not known	

MGMT promotor methylation status was assessed via methylation-specific, quantitative real-time polymerase chain reaction (PCR), following bisulfite treatment on isolated DNA acquired from formalin-fixed, paraffin-embedded tumor samples. IDH-mutation status was detected using immunohistochemistry labeling followed by IDH hotspot sequencing. Digital image analysis was applied to quantify the Ki-67 proliferation index.

Patients were classified into three groups of equal size based on the timing of initiation of RT (i.e. early, regular, late initiation of RT). Patients that started their RT before the 28th day after surgery represented the early group, those between the 28th and 33rd the regular group, and the ones after day 33 the late group.

Survival was also evaluated in a small subset of patients where RT was applied either very early or with a significant delay. Patients were stratified into three groups (RT before 14 days, RT between 14 and 48 days and RT after 48 days). Our very early time frame was chosen on the basis of data from Peker et al., who showed that starting RT prior to the 14th postoperative day causes significantly higher levels of tissue damage compared to commencing radiation treatment after 3 weeks or more [20]. The cutoff point that defined very late initiation of RT (very late group) was set at 48 days, since this represents the point of time when tumor cells have been duplicated twice [21]. Patients that began their RT between those two cutoff points represented our regular group.

For all patients a preoperative and a postoperative contrast-enhanced (CE) magnetic resonance imaging (MRI) study was obtained no later than 48 h after surgery. The imaging parameters analyzed included tumor location, involvement of midline structures (as seen in FLAIR sequences), preoperative tumor volume (PTV) and RTV. Tumor volume was defined as the area of increased signal intensity on contrast-enhanced (CE)-T1w images (including any necrotic areas). RTV was assessed on CE-T1 digitally subtracted sequences. The subtraction involved high signal areas on postoperative, precontrast images from postoperative, postcontrast images. For each axial MRI-slice the tumor margins were traced using the iPlan software (BrainLab, Germany). Volumes were calculated as the product of the area traced and the corresponding slice thickness. The sum of each slice volume gave the tumor volume in cubic mm. All the measurements were performed independently by a neuroradiologist and a neurosurgeon whose results were blind to each other’s.

Follow-up consisted of clinical examination and MRI every 3 months or upon clinical deterioration. The primary endpoints were overall survival (OS) defined as time from first surgery until death or the end of follow-up and progression-free survival (PFS) defined as time from surgery until first progression. Tumor progression was defined using the RANO criteria [27].

### Statistical analysis

Quantitative baseline patient characteristics are presented as median, except age which is presented as mean ± standard deviation. Categorical variables are provided in terms of counts and percentages. Differences between the cohorts were evaluated using the Median test for quantitative characteristics, the independent t-test for age and the Chi-Square test for categorical variables. OS and PFS were estimated using the Kaplan-Meier analysis and the log-rank test was used for group comparison. KPS and Ki-67 were treated as dichotomous variables with the cut-off point set at 70 and 15, respectively [28].

Pearson correlation coefficient was used to evaluate the interrater reliability.

Probability (p) values of less than 0.05 were considered to be statistically significant.

A Cox proportional hazards regression model was used for univariate analysis to test the effect of all potential prognostic factors in terms of survival. Variables exibiting statistical significance on univariate analysis were then included in a multivariate logistic regression model. This model allows the identification of important prognostic factors. Hazard ratios (HR) with a 95% confidence interval (CI) were calculated.

Statistical analyses were performed using the Statistical Package for Social Sciences (SPSS, version 24, Chicago, IL, USA).

## Results

### Patient population

A total of 151 patients met the inclusion criteria. Their clinical, radiographic and tumor characteristics are summarized in Table 2. The total population comprised 96 men (64%) and 55 women (36%) with a mean age of 60.3 years (SD 11.9 years). The median preoperative KPS (range 20–100) was 90. Upon comparing the patient characteristics among the three cohorts no statistical significant difference was observed (Table 2).

**Table 2:** Patient characteristics (Terciles)

Characteristics	Total	< 28 Days (early *n* = 51)	28–33 Days (regular *n* = 52)	> 33 Days (late *n* = 48)	*p* Value
Age, y^a^	60.3+/−11.9	61.4+/−7.8	60.2+/−12.1	62.2+/−12.1	0.964
Male	96(64%)	30 (59%)	31(60%)	35(73%)	0.265
KPS^b^	90	90	90	90	0.521
KPS < 70	11(7.3%)	3(6%)	5(10%)	3(6%)	0.725
Preoperative symptoms					
Duration of symptoms, days^b^	3(1–48)	4(1–40)	3(1–48)	3(1–48)	0.736
Headache	52(34%)	15(29%)	22(42%)	15(31%)	0.331
Nausea/vomiting	18(12%)	5(10%)	7(14%)	6(13%)	0.839
Motor deficit	71(47%)	28(55%)	24(46%)	19(40%)	0.308
Sensory deficit	16(11%)	9(18%)	4(8%)	3(6%)	0.129
Seizures	54(36%)	20(39%)	19(37%)	15(31%)	0.703
Language deficit	54(36%)	20(39%)	20(39%)	14(29%)	0.512
Visual impairment	28(19%)	7(14%)	9 (17%)	12(25%)	0.339
Ataxia/gait deficit	58(38%)	20(39%)	19(37%)	19(40%)	0.942
Cognitive deficit	72(48%)	24(47%)	28 (54%)	20(42%)	0.473
Altered level of consciousness	5(3%)	0(0%)	3(6%)	2(4%)	0.242
Radiographic characteristics					
Midline structures involvement (FLAIR)	22(15%)	8(16%)	9(17%)	5(10%)	0.598
Left hemisphere	74(49%)	24(47%)	23(44%)	27(56%)	0.458
Preoperative tumor volume, cm^3b^	22.4(1.4-	24.8(1.4-	20.4(2.3–122.7)	18.7(1.5–96)	0.769
	122.7)	112.7)			
Residual tumor volume, cm^3b^	0.1(0–13.1)	0.5 (0–11.8)	0.1 (0–7.9)	0(0–13.1)	0.128
Extent of resection^b^	100%(32.7-	99%(73-	100%(33–100%)	100%(79-	0.101
	100)	100%)		100%)	
Miscellaneous					
Duration of hospitalization, days^b^	8(3–40)	8(5–29)	7(3–18)	9(4–40)	0.017
Duration of the operation, min^b^	222(94–524)	206(94–524)	224(100–405)	225(140-	0.584
				419)	
Ki-67^b^	20	30	20	20	0.840
Ki-67 < 15	23(15%)	7(14%)	10(19%)	6(13%)	0.622
MGMT negative	89(59%)	31(62%)	34(65%)	24(51%)	0.322
Survival, months^c^	17.3(13.7–20.9)	15(11.5–18.5)	17.4(10.7–24.1)	18,2(12.9–23.5)	0.902

^a^Mean ± SD

^b^Median

^c^Kaplan-Meier estimate of median postoperative survival

### Interobserver agreement

Interobserver agreement for pre- and postoperative volume measurements was very high in all cases (r_s_ > 0.95).

### Progression-free survival

**Fig. 1:**
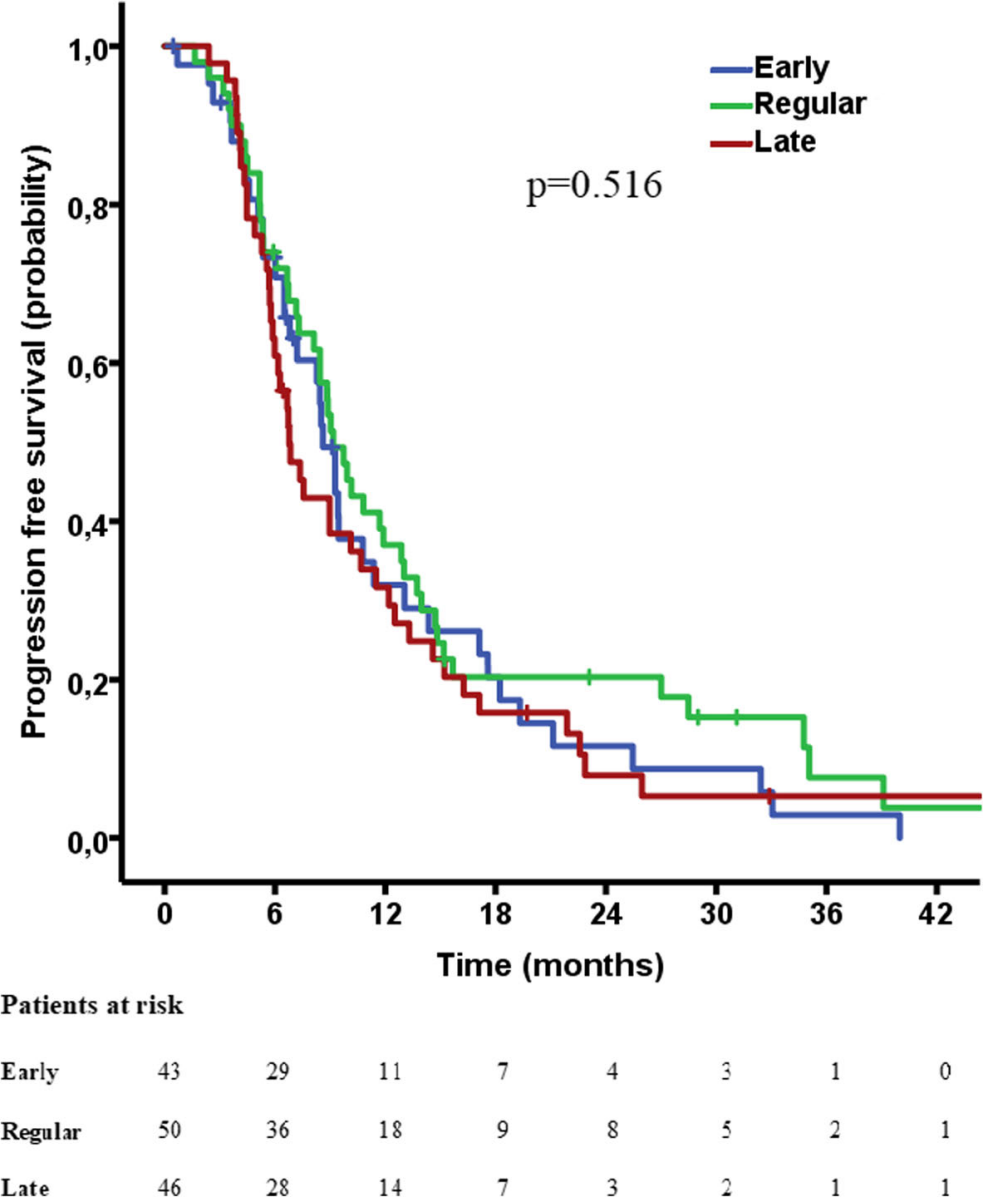
Kaplan-Meier curve for PFS between the three groups, showing no difference in PFS between the groups (*p* = 0.728) . Hash marks indicate censored cases

Median PFS for the “early” group was 8.6 months (95% CI 7.5–9.7 months), for the “regular” 9.2 months (95% CI 7.7–10.7 months) and for the “late” 6.8 months (95% CI 5.4–8.1 months). No statistical significance between the groups was reached (*p* = 0.516) (Fig. 1).

Significant predictors of PFS determined by the univariate analysis included midline structures involvement, EOR and RTV (Table 3). Midline structures involvement and RTV were identified as independent prognostic factors for PFS in the multivariate analysis (Table 4).

**Table 3:** Univariate Cox regression analysis for PFS

Variable	HR (95% CI)	*p* Value
Age	1.01(1.00–1.02)	0.187
Gender	1.34(0.92–1.94)	0.128
KPS	1.00(0.98–1.01)	0.733
KPS < 70	0.84(0.41–1.73)	0.639
Duration of symptoms	1.02(1.00–1.04)	0.108
Headache	0.86(0.60–1.25)	0.438
Nausea/vomiting	1.01(0.58–1.77)	0.967
Motor deficit	1.02(0.72–1.45)	0.915
Sensory deficit	0.66(0.35–1.23)	0.187
Seizures	1.05(0.73–1.51)	0.796
Language deficit	0.98(0.68–1.43)	0.926
Visual impairment	0.92(0.58–1.46)	0.728
Ataxia/gait deficit	0.94(0.65–1.35)	0.733
Cognition deficit	1.01(0.71–1.44)	0.967
Altered level of consciousness	0.71(0.29–1.74)	0.453
Midline Structures Involvement	2.80 (1.67–4.74)	< 0.001
Left hemisphere	0.90(0.63–1.28)	0.549
Preoperative tumor volume	1.00(0.99–1.01)	0.657
RTV	1.17(1.09–1.26)	< 0.001
EOR	0.97(0.95–0.99)	0.001
Duration of hospitalisation	1.00(0.95–1.04)	0.835
Duration of the operation	1.00(1.00–1.00)	0.885
Ki-67	1.00(0.99–1.01)	0.910
Ki-67 < 15	1.32(0.78–2.23)	0.309
MGMT	1.35(0.93–1.94)	0.111

**Table 4:** Multivariate Cox regression analysis for PFS

Variable	HR (95% CI)	*p* Value
Midline Structures Involvement	2.84(1.41–5.74)	0.004
RTV	1.11(1.02–1.22)	0.021
EOR	0.99(0.96–1.01)	0.273

### Overall survival

**Fig. 2:**
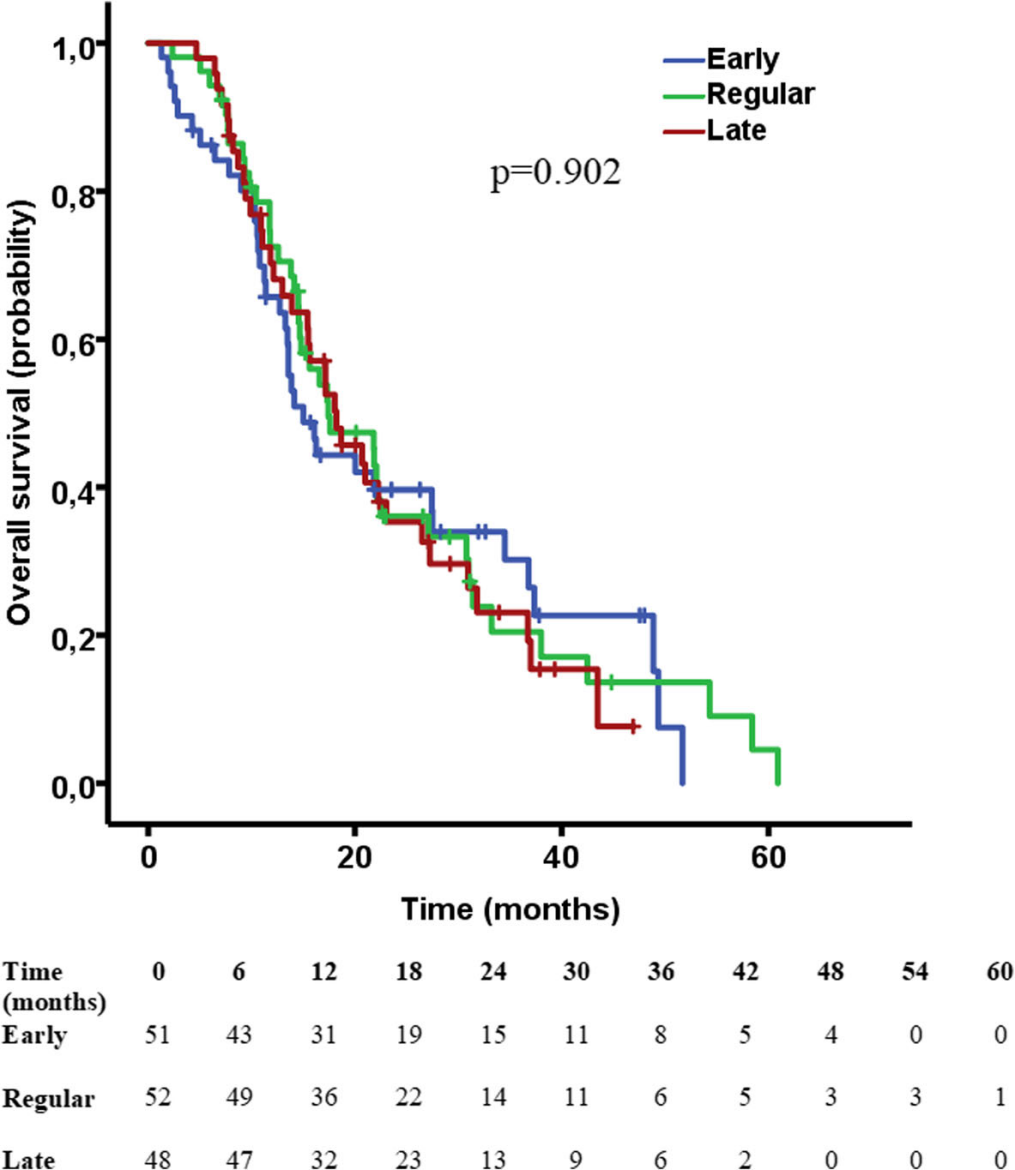
Kaplan-Meier curve for OS between the three groups, showing no difference in OS between the groups (*p* = 0.902). Hash marks indicate censored cases

Median OS for the “early” group was 15.0 months (95% CI 11.5–18.5 months), for the “regular” 17.4 months (95% CI 10.7–24.1 months) and for the “late” 18.2 months (95% CI 12.9–23.5 months). The differences between groups were not significant (p = 0.902) (Fig. 2).

The results of the the univariate analysis are reported in Table 5. Age, duration of symptoms, midline structures involvement, RTV, MGMT and Ki67 were identified as significant prognostic factors. These factors were included in our multivariate regression model. Age, midline structures involvement, RTV, MGMT and Ki67 were identified as independent prognostic factors for survival (Table 6).

**Table 5:** Univariate Cox regression analysis forOS

Variable	HR (95% CI)	*p* Value
Age	1.03(1.01–1.04)	0.001
Gender	0.62(0.42–0.92)	0.019
KPS	1.00(0.98–1.02)	0.872
KPS < 70	0.89(0.43–1.88)	0.765
Duration of symptoms	1.02(1.00–1.04)	0.041
Headache	0.78(0.53–1.15)	0.210
Nausea/vomiting	0.85(0.48–1.50)	0.594
Motor deficit	0.94(0.65–1.37)	0.763
Sensory deficit	0.69(0.35–1.36)	0.280
Seizures	0.86(0.58–1.26)	0.432
Language deficit	0.93(0.62–1.38)	0.703
Visual impairment	1.17(0.73–1.88)	0.520
Ataxia/gait deficit	0.78(0.52–1.16)	0.214
Cognition deficit	1.03(0.71–1.50)	0.866
Altered level of consciousness	0.51(0.16–1.65)	0.258
Midline Structures Involvement	2.52(1.50–4.23)	< 0.001
Left hemisphere	0.94(0.65–1.37)	0.760
Preoperative tumor volume	1.00(0.99–1.01)	0.837
RTV	1.08(1.01–1.16)	0.023
EOR	0.99(0.97–1.01)	0.215
Duration of hospitalisation	1.04(1.00–1.08)	0.084
Duration of the operation	1.00(1.00–1.00)	0.260
Ki-67	1.01(0.99–1.02)	0.400
Ki67 < 15	1.93(1.03–3.60)	0.039
MGMT	1.72(1.55–2.57)	0.008

**Table 6:** Multivariate Cox regresison analysis for OS

Variable	HR (95% CI)	*p* Value
Age	1.02(1.01–1.04)	0.015
Midline Structures Involvement	4.23(2.04–8.76)	< 0.001
RTV	1.12(1.04–1.22)	0.005
MGMT	2.07(1.29–3.32)	0.002
Ki67	3.16(1.55–6.44)	0.002
Duration of symptoms	1.02(0.99–1.05)	0.156
Gender	0.64(0.41–1.02)	0.058

### Survival analysis of the extremes of timing of RT

**Fig. 3:**
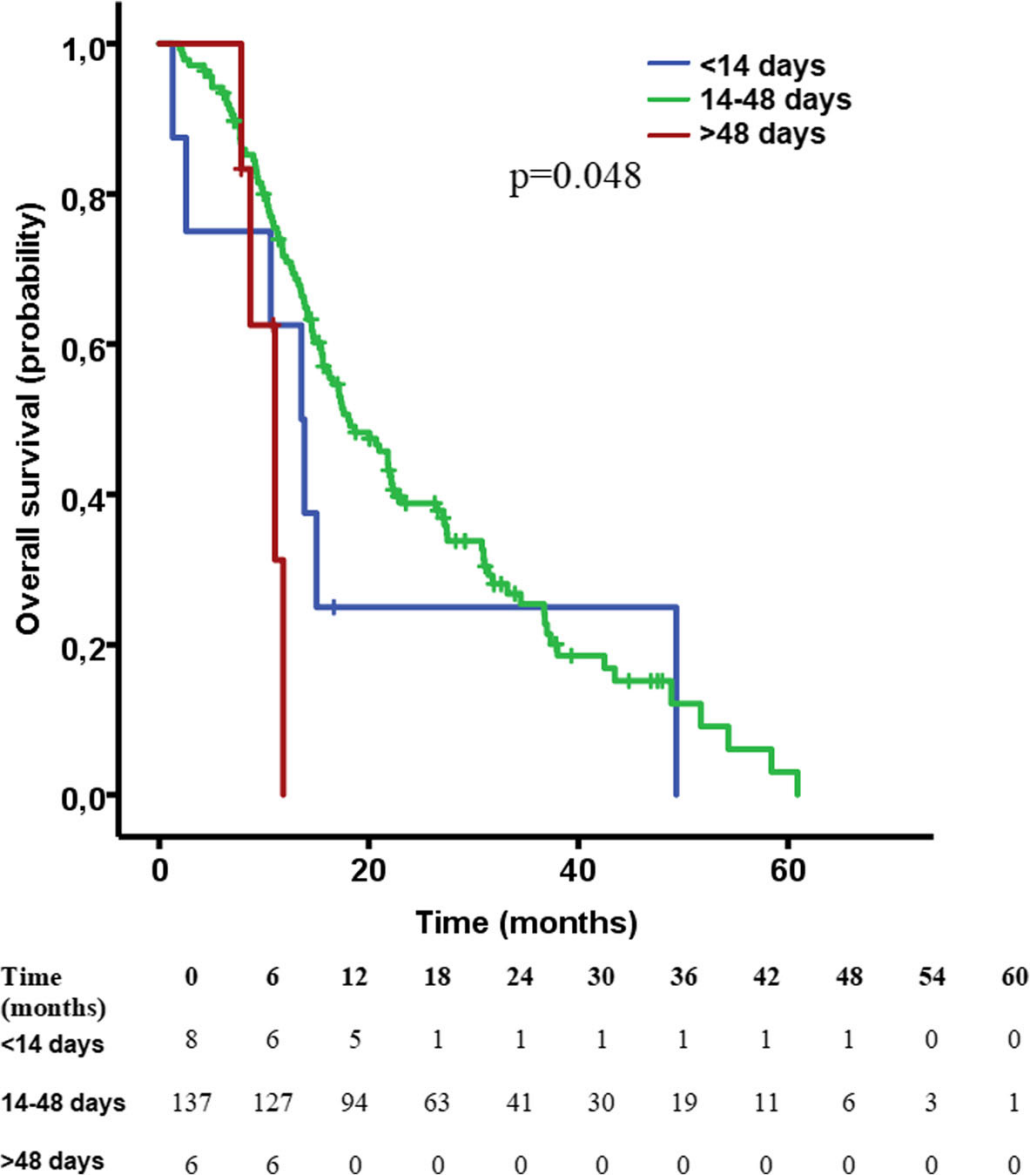
Kaplan-Meier curve for OS between the three groups, showing significantly longer survival for the “regular group” compared with the “very late group” of patients (*p* = 0.034). Hash marks indicate censored cases

Log rank analysis of OS exhibited significant differences between groups (*p* = 0.048, Fig. 3 right). OS was significantly longer for the “regular group” (18 months, 95% CI: 13.8–22.2 mo) compared with the “very late group” of patients (11 months, 95% CI: 7.4–14.7 mo, *p* = 0.034). Patients starting radiochemotherapy within 15 days had a median OS of 13.6 months (95% CI: 9.1–18 mo), which was less than the “regular group” but statistical significance was not reached (HR = 1.59, *p* = 0.239). A delay of more than 48 days in the initiation of RT was found to be an independent prognostic factor of OS in multivariate Cox regression analysis (*p* = 0.003).

## Discussion

The effect of initiation of RT on survival of glioblastoma patients has been a matter of debate for a long time.

The possible detrimental effect of delayed RT on survival could be partially related to the tumor cell biology. Tumor growth rate is best described by the Gompertz Sigmoid curve, which means that the rate slows down as the tumor size increases [29, 30]. Since radiosensitivity decreases as the tumor growth rate falls, late initiation of RT could lead to reduced effectiveness of radiation [21]. Another possible explanation is that it is likely that after 48 days a certain amount of tumor has regrown, particularly when residual tumor is left in situ. This residual tumor burden of a fast growing tumor could negate the positive effects of an i.e. near-total resection, explaining the worse OS.

On the other hand early start of RT has also been linked with a negative impact on survival: from a physiological point of view hypoxia and edema surrounding the surgical bed in the early postoperative period may decrease radiosensitivity [19]. Moreover the surgical cavity has not shrunk substantially, leading to an increased radiation field and thus to increased normal tissue damage [31]. Increased damage to normal brain tissue with initiation of RT within 2 weeks postoperatively has been demonstrated in an animal model [20]. Increased damage to normal brain tissue could lead to delayed recovery, a worse clinical condition and subsequently to a shorter OS.

In the era before the introduction of TMZ studies of the field provided controversial results. Burnet et al. introduced a mathematical model that predicts poorer outcome after delay to start RT. No patient survived long-term after a 70-day delay [21]. Two retrospective studies of patients with grade III/IV gliomas found that the risk of death increased by 8.9% per week and by 2% per day of waiting for RT [32, 33]. Their results were contradicted by other studies that showed no detrimental effect of delay of RT initiation on survival [19, 34]. The longest series with 2855 patients based on the prospective trials of the Radiation Therapy Oncology Group (RTOG) demonstrated that delaying RT up to 6 weeks after histological confirmation of the tumor did not reduce survival. It did however show a significantly decreased survival of early (< 2 weeks) initiation of RT [19]. Another large study with 1375 elderly patients based on the Surveillance, Epidemiology, and End Results (SEER) Medicare database concluded that the timing of cranial radiation had no impact on survival [34]. However, in those studies concomitant and adjuvant TMZ was not yet standard-of-care for first line treatment of GBM, making their results difficult to interpret.

In the modern era of standard combined radiochemotherapy the results remained controversial. A study with 2535 patients with high grade glioma (HGG) based on the Clinformatics Data Mart database demonstrated a significant decrease in survival for early initiation of RT [35]. Alnaami et al., Adeberg et al., and Han et al. concluded a similar effect of early initiation of RT, while in the studies of Wang et al. and Wehming et al. initiation of RT could not be identified as an independent prognostic factor [15–17, 36, 37]. Three studies found no significant impact of waiting time to RT on survival [18, 38, 39]. In their retrospective study of 12,738 patients, Pollom et al. found a survival benefit for patients that had their RT initiated within 35 days following gross total resection [40]. Other studies also presented a detrimental effect of longer delays on OS [14, 22, 41, 42].

All those studies suffer from significant biases such as an inhomogeneous patient population in terms of tumor type and therapeutical protocols. No study took into consideration the IDH mutation status, so according to the 2016 WHO classification criteria by definition their populations probably included both primary and secondary GBM [23]. The studies of Wehming et al. and Nathan et al. included GBM as well as Anaplastic Astrocytomas [35, 37]. In the study of Louvel et al. a number of patients had carmustine wafer implantation, and in that of Randolf et al. a proportion of the patient population underwent biopsy [38, 43].

Very few studies controlled for EOR and none for RTV, which seems to be an even more significant predictor of survival than EOR [44]. 3-D volumetric analysis was not performed in any of the studies that controlled for EOR, which is necessary for the accurate evaluation of EOR as a prognostic factor. The relationship between EOR and RT, if any, is unclear. The intiutive hypothesis would be that a patient with significant RTV would benefit from early initiation of RT while a patient without RT could theoreticaly delay RT, but this hypothesis could not be validated in the various studies. In the study by Pollom et al., the observed survival benefit after early RT was found only for patient without RTV, while patients with residual tumor did not experience any survival benefit. Similarly, Valduvieco et al. found improved survival with earlier initiation of RT in patients who got gross total resection [42]. It seems that EOR is an independend prognostic factor and given the short doubling time of GBM, residual tumor may be a far more dominant factor than early initiation of RT. Our results confirm this hypothesis: RTV was an independend prognostic factor for both OS and PFS.

In the literature, only a few studies report on performance data, or controlled for tumor eloquency. Moreover, only two studies included the MGMT methylation status: Adeberg et al. demonstrated a negative impact of initiation of RT earlier than 24 days, but only 50 patients were included, while Spratt et al. showed a survival detriment with delaying RT post-surgery for more than 6 weeks, but the MGMT status was determined in only 45.8% of the entire cohort [15, 22]. Finally, no study took into consideration the Ki-67 proliferation index.

In our study we made an effort to minimize such biases by studying a more homogeneous population in terms of tumor type and oncological treatment. For example, we included only newly diagnosed primary GBM, non-IDH-1 mutated forms, that were treated with surgery with the goal of gross total resection of the tumor followed by radiochemotherapy according to the EORTC 22981/26981 protocol while taking into account the MGMT promotor methylation status. We also checked for EOR and RTV using a precise quantitative method. We included the MGMT promotor methylation status, which represents an established prognostic factor, and the Ki-67 proliferation index, which is one of the most studied biomarkers of GBM. Additionally, our groups were very similar in respect to all important demographic, clinical and molecular factors. This is important since it allows us to assimilate reliable results when comparing the groups on survival analysis. It also shows that a patient-selection bias could be, to a certain extent, avoided.

Upon comparing the three patient groups (early, regular, late), timing of RT failed to exhibit a statistically significant impact on PFS or OS. The independent predictors of PFS include midline structures involvement and RTV, while age, midline structures involvement, RTV, MGMT and Ki67 were identified as independent prognostic factors of OS. Our results agree with the majority of studies conducted in the modern era were the Stupp protocol is applied [15–18, 36–39].

Since several studies suggested that there is a possible impact on survival when starting RT prematurely or with a significant delay, and although not our primary analysis goal due to important biases, we also conducted a survival analysis for the small subsets of patients that received RT earlier than 14 days or after 48 days postoperatively [14, 16, 22, 35, 41, 42]. A significantly shorter OS was shown for the patients that started RT after a prolonged delay. Patients initiating RT very early also had a shorter OS but the difference was not significant. Whether this is a true effect of the timing of radiation or an epiphenomenon of the fact that these patients were in no condition to receive RT earlier (which is a poor prognostic factor by itself) is unclear.

A limitation met here was the small number of patients on the “very early” and “very late” groups, thus undermining the statistical power of our analysis. Another major issue that arises in our study, as well as in all the studies that determined timing of RT as an independent prognostic factor, is the lack of data regarding the reasons for the delayed or the early start of RT. Therefore it cannot be assessed whether the reduced survival observed was indeed due to the timing of RT or due to comorbid conditions. An example would be a patient with a large residual tumor who is rushed to RT, or a patient with a reduced postoperative KPS who needs to recover before receiving RT. Another important issue, is the fact that the OS is affected not only by the primary treatment, but possibly also by the further treatment that the patients received after progression. The effect of the strategy followed at progression could be significant, but it cannot be isolated. Furthermore our study is inherently limited because of its retrospective nature. As such it suffers shortcomings such as selection and clinical data bias, lack of randomization, and a cause and effect conclusion cannot be established. Prospective, randomized clinical trials are required to validate the effect of timing of RT on survival, although ethical concerns render such an endeavor challenging.

## Conclusions

Timing of RT within a timeframe of up to 48 days postoperatively does not appear to be associated with worsened survival. A delay beyond this timeframe may be associated with worse OS. Our results further support the notion that RT should start promptly, as soon as the patient has recovered from surgery. Determining the optimal timeframe between surgery and RT merits further investigation.

## Abbreviations

CE: Contrast-enhanced MRI: Magnetic resonance imaging
GBM: Glioblastoma RT: Radiotherapy TMZ: Temozolomide
IDH: Isocitrate dehydrogenase PCR: Polymerase chain reaction
KPS: Karnofky performance score EOR: Extent of tumor resection
PTV: Preoperative tumor volume OS: Overall survival PFS: Progression free survival
RTV: Residual tumor volume MGMT: O-6-Methylguanine Methyltransferase
